# Genetic variants in 3′-UTRs of methylenetetrahydrofolate reductase (*MTHFR*) predict colorectal cancer susceptibility in Koreans

**DOI:** 10.1038/srep11006

**Published:** 2015-06-05

**Authors:** Young Joo Jeon, Jong Woo Kim, Hye Mi Park, Jung O Kim, Hyo Geun Jang, Jisu Oh, Seong Gyu Hwang, Sung Won Kwon, Doyeun Oh, Nam Keun Kim

**Affiliations:** 1 Institute for Clinical Research, School of Medicine, CHA University, 351 Yatap-dong, Bundang-gu, Seongnam 463-712, South Korea; 2 Department of Surgery, School of Medicine, CHA University, 351 Yatap-dong, Bundang-gu, Seongnam 463-712, South Korea; 3 Department of Internal Medicine, School of Medicine, CHA University, 351 Yatap-dong, Bundang-gu, Seongnam 463-712, South Korea

## Abstract

Polymorphisms in the methylenetetrahydrofolate reductase (*MTHFR*) play important roles in tumor development, progression, and metastasis. Moreover, recent studies have reported that a number of 3′-UTR polymorphisms potentially bind to specific microRNAs in a variety of cancers. The aim of this study was to investigate the association of four *MTHFR* polymorphisms, 2572C>A [rs4846049], 4869C>G [rs1537514], 5488C>T [rs3737967], and 6685T>C [rs4846048] with colorectal cancer (CRC) in Koreans. A total of 850 participants (450 CRC patients and 400 controls) were enrolled in the study. The genotyping of *MTHFR* 3′-UTR polymorphisms was performed by polymerase chain reaction-restriction fragment length polymorphism analysis or TaqMan allelic discrimination assay. We found that *MTHFR* 2572C>A, 4869C>G, and 5488C>T genotypes were substantially associated with CRC susceptibility. Of the potentially susceptible polymorphisms, *MTHFR* 2572C>A was associated with increased homocysteine and decreased folate levels in the plasma based on *MTHFR* 677CC. Our study provides the evidences for 3′-UTR variants in *MTHFR* gene as potential biomarkers for use in CRC prevention.

Colorectal cancer (CRC) is the third most common type of cancer and the second leading cause of cancer-related mortality in Western countries^1^. The prognosis of CRC patients is dependent on the tumor stage at the time of diagnosis. However, over 57% of patients have regional or distant spread of cancer cells at the time of diagnosis^2^. The pathogenesis of CRC usually follows a stepwise progression from benign adenoma to invasive adenocarcinoma^3^. In colorectal carcinogenesis, the unique molecular and genetic alterations that occur within cells result in a specific CRC phenotype^4^. This phenotype is connected to variable tumor behaviors that are relevant to the prognosis and the response to specific therapies^4^. As a result, the term “CRC” no longer refers to a single disease, but rather a heterogeneous group of diseases associated with a diversity of genetic/epigenetic backgrounds. In this respect, many ongoing studies are aimed at evaluating biomarkers as potential predictors of prognosis or response to therapy, which will most likely lead to the individualized management of the disease.

Folate (FA) is important for cell division and homeostasis due to its essential role in the synthesis of S-adenosyl-methionine, the methyl donor required for all methylation reactions in the cell. In addition to its function in cell homeostasis, FA has been hypothesized to play a role in carcinogenesis, especially in development of CRC^5^. Several mechanisms could underlie FA deficiency-mediated CRC, including DNA strand breaks, aberrant DNA methylation, and impaired DNA repair. Thus, FA has been proposed as a possible candidate nutrient for CRC prevention^6^. Genetic variants in FA metabolism-related genes may modulate levels of this vitamin and influence risk of carcinogenesis. Furthermore, previous meta-analyses of numerous epidemiologic studies reported that FA was a determinant of CRC risk^7^.

The effect of several polymorphic genes involved in FA metabolism, including methylenetetrahydrofolate reductase (*MTHFR*) on CRC susceptibility and progression has been investigated^8^ MTHFR catalyzes the reduction of 5,10-methylenetetrahydrofolate to 5-methyltetrahydrofolate; the latter is the methyl donor for the conversion of homocysteine (Hcy) to methionine, whereas the former, and its derivatives, are essential cofactors for both thymidylate and *de novo* purine synthesis^9^,^10^. During *de novo* purine synthesis, thymidylate synthase with the FA binding site catalyzes the conversion of deoxyuridine monophosphate (dUMP) to deoxythymidine monophosphate (dTMP). This conversion is indispensable for the production of thymine, a nucleotide needed for DNA synthesis and repair^11^,^12^. Decreased MTHFR activity and expression lead to an accumulation of Hcy and/or deficiency of FA^13^.

There are two well-studied polymorphisms of the *MTHFR* gene, i.e., *MTHFR* 677C>T and 1298A>C. Despite the existence of a large body of data for studies of associations between these polymorphisms and CRC, findings of genetic associations have been inconsistent for a variety of conditions^8^. Recent studies have shown some clinical impacts of polymorphisms in the 3′-UTR of certain genes, which may potentially bind to specific microRNAs (miRNAs) in various cancers^14^,^15^,^16^,^17^. However, variants in the *MTHFR* 3′-UTR have not been extensively studied.

In the present study, four single nucleotide polymorphisms (SNPs) in the *MTHFR* 3′-UTR were identified by a database search; these are *MTHFR* 2572C>A (rs4846049), 4869C>G (rs1537514), 5488C>T (rs3737967), and 6685T>C (rs4846048). There were 17 *MTHFR* 3′-UTR SNPs with minor allele frequencies >5% in the global population. The 13 SNPs in the *MTHFR* 3′-UTR were excluded from this study due to following reasons: (1) lack of information for validation in the Asian population (rs35134728 and rs55780505), (2) below 5% of minor allele frequency in the Asian population (rs2184226, rs868014, rs2077360, and rs4845884), and (3) failure of genotyping conditions (rs1537516, rs1537515, rs2184227, rs3737966, rs3820192, rs11559040, and rs72640221). The minor allele frequencies of the four SNPs were all >5% in the Asian population. Little is known about their genetic associations with CRC. Therefore, we investigated whether these polymorphisms of the *MTHFR* 3′-UTR correlate with CRC susceptibility in Koreans.

## Results

### Genetic susceptibility of single and multiple markers

Table 1 presents demographic characteristics between cases and controls. Table 2 shows the distributions of genotypes and haplotypes for *MTHFR* 3′-UTR polymorphisms (2572C>A, 4869C>G, 5488C>T, and 6685T>C) in CRC patients and control subjects. The genotype frequencies of controls and patients were consistent with expectations under Hardy-Weinberg equilibrium (HWE). Supplementary Table S1 shows the minor allele frequencies of *MTHFR* 3′-UTR polymorphisms described in previous reports. The allele frequencies of the control group in this study were similar to former results. *MTHFR* 2572CA + AA [adjusted odds ratio (AOR) = 1.49, 95% confidence interval (CI) = 1.10–2.03, *P* = 0.010], *MTHFR* 4869CG + GG (AOR = 2.17, 95% CI = 1.41–3.33, *P* < 0.001), and *MTHFR* 5488CT + TT (AOR = 1.66, 95% CI = 1.13–2.42, *P* = 0.001) were significantly associated with CRC susceptibility. The statistical significances remained after false discovery rate (FDR) correction by Benjamini-Hochberg method. Supplementary Table S2 shows AOR values of *MTHFR* 3′-UTR genotypes according to confounding variables. There were variations of AOR values for *MTHFR* 3′-UTR genotypes, but the statistical significances were unchanged for all conditions. To evaluate combined effects of *MTHFR* 3′-UTR SNP loci on CRC incidence, logistic regression for the combined genotypes and haplotypes was performed. The 2572CA + AA/4869CG + GG (AOR = 2.09, 95% CI = 1.35–3.24*, P* < 0.001), 4869CG + GG/5488CT + TT (AOR = 2.12, 95% CI = 1.38–3.27, *P* < 0.001), and 2572A-4869G-5488T-6685T (AOR = 1.99, 95% CI = 1.31*–*3.00, *P* = 0.001) types contributed to CRC prevalence (Table 3). However, the combined alleles for *MTHFR* 3′-UTR SNP loci did not show much stronger genetic associations than single genotypes.

### Stratified effects of clinical and environmental factors

Unlike other cancers, CRC epidemiology was affected by a variety of identified risk factors in a complex manner^3^. Previous reports identified the following risk factors: aging, male gender, obesity, metabolic syndrome (MetS), hypertension (HTN), diabetes mellitus (DM), deficiency of FA, increased intake of red meat, excessive alcohol consumption, and smoking^3^,^18^,^19^,^20^. In addition, epidemiologic alterations of CRC pathology and genetic differences correlating with the clinical features of CRC have been previously reported^21^,^22^,^23^. Therefore, stratified analyses were useful in elucidating CRC epidemiology resulting from a diversity of confounding variables. Except for FA deficiency, which presented difficulties in establishing a threshold using plasma levels, we conducted stratified analyses of the data according to age, gender, tumor site, tumor size, tumor node metastasis (TNM) stage, presence of MetS, HTN, DM, levels of body mass index (BMI), triglycerides (TG), and high density lipoprotein-cholesterol (HDL-C) to determine whether the 3′-UTR minor alleles were associated with CRC incidence in specific subsets of the study population. The results for the recessive model were excluded because of a small number of 3′-UTR minor homozygous genotypes when stratified by a variety of factors. FDR correction was used to eliminate false positive associations from stratified effects. Supplementary Table S3 summarizes the frequencies of *MTHFR* 3′-UTR genotypes in each stratified CRC group. The overall results of stratified analyses are shown in Table 4. *MTHF*R 2572CA + AA presented subset-specific associations in subgroups of patients with the following: ≥62 years of age (AOR = 1.72), male (AOR = 2.52), rectal cancer (AOR = 1.89), tumors ≥5 cm (AOR = 1.75), TNM stage I/II disease (AOR = 1.70), HTN (AOR = 2.03), DM (AOR = 2.03), and ≥150 mg/dL of TG (AOR = 2.20). *MTHFR* 4869CG + GG displayed subset-specific associations in subgroups of patients with the following: male (AOR = 5.05), rectal cancer (AOR = 2.70), tumors ≥5 cm (AOR = 2.64), TNM stage I/II disease (AOR = 2.33), HTN (AOR = 2.32), DM (AOR = 2.55), <25 kg/m^2^ of BMI (AOR = 2.12), ≥150 mg/dL of TG (AOR = 4.20), and ≥40 (male)/50 (female) mg/dL of HDL-C (AOR = 2.59). *MTHFR* 5488CT + TT showed subset-specific associations in subgroups of patients with the following: ≥62 years of age (AOR = 1.96), male (AOR = 2.99), rectal cancer (AOR = 2.02), tumors ≥5 cm (AOR = 2.02), TNM stage I/II disease (AOR = 1.84), HTN (AOR = 2.42), <25 kg/m^2^ of BMI (AOR = 2.12), and ≥40 (male)/50 (female) mg/dL of HDL-C (AOR = 1.86).

### Combined effects of 3′-UTR polymorphisms with environmental factors

Because cancer risk is determined by the complex interplay of genetic and environmental factors, we calculated combined gene-environment effects on CRC susceptibility (Table 5 and Supplementary Tables S4–S7). To analyze combined gene-environment effects, we chose the following environmental risk factors which were significantly prevalent in the CRC group: lower plasma FA levels, MetS, HTN, DM, and <40 (male)/50 (female) mg/dL of HDL-C. We used the relative excess odds due to interaction (RERI_OR_) to evaluate the additivity of odds between 3′-UTR genotypes and environmental risk factors^24^. With RERI_OR_ >0, there was an additive interaction between the combined factors. All *MTHFR* 3′-UTR minor genotypes (2572CA + AA, 4869CG + GG, 5488CT + TT, and 6685TC +CC) showed RERI_OR_ >0 in combinations with <5.77 ng/mL of plasma FA (the lowest tertile interval), MetS, and HTN (Table 5, Supplementary Tables S4 and S5). The 6685TC + CC genotype showed RERI_OR_ <0 in combination with DM, whereas 4869CG + GG and 5488CT + TT genotypes showed RERI_OR_ <0 in combination with lower HDL-C levels (Supplementary Tables S6 and S7). *MTHFR* 2572C>A was identified as the best polymorphism, showing additive interactions with the chosen environmental risk factors.

### Variations of genetic associations for 2572C>A by 677C>T genotypes

Next, we sought to determine whether the polymorphisms of interest within the 3′-UTR of the *MTHFR* gene correlated with plasma Hcy and FA concentrations (Table 6). We analyzed plasma Hcy and FA levels according to studied 3′-UTR polymorphisms based on *MTHFR* 677C>T, due to its strong associations with MTHFR activity^13^. The CC genotypes of *MTHFR* 677C>T and *MTHFR* 2572C>A showed significant correlations with increased plasma Hcy (regression coefficient = 1.120, *t* value = 3.361, *P* = 0.001) and decreased plasma FA (regression coefficient = −1.370, *t* = −2.595, *P* = 0.010) levels. *MTHFR* 6685T>C showed a correlation with decreased plasma FA (regression coefficient = −1.482, *t* = −2.428, *P* = 0.016) levels with the 677CC type. We also quantified expression of MTHFR mRNA in 47 tumor and tumor-adjacent tissue samples and searched for differences in expression based on the *MTHFR* genotype. The characteristics of 47 tumor and tumor-adjacent tissues are shown in Supplementary Table S8. The *MTHFR* 2572A allele had a tendency of decreased MTHFR mRNA expression [presenting lower –delta (Δ) cycle of threshold (C_T_) values derived from the equation as –ΔC_T_ = –(C_T MTHFR_ – C_T 18S rRNA_)] in tumor-adjacent tissues (Supplementary Table S9). Supplementary Table S10 shows MTHFR mRNA expression of 2572C>A based on the 677C>T genotypes. We excluded data for 4869C>G, 5488C>T, and 6685T>C due to a small number of minor genotypes not reaching three individuals with stratified 677C>T genotypes. Similar to the results of Table 6, *MTHFR* 2572CA + AA displayed lower –ΔC_T_ values of mRNA expression within the 677CC genotype. Because the functionality of *MTHFR* 2572C>A was affected by the 677C>T genotypes, we examined AOR values of 3′-UTR minor genotypes when stratified by 677C>T (Supplementary Table S11). *MTHFR* 2572CA + AA, 4869CG + GG, and 5488CT + TT increased AOR values for CRC prevalence within the CT + TT genotype of 677C>T.

## Discussion

In the present study, we investigated whether four 3′-UTR polymorphisms of the *MTHFR* gene are related to the occurrence of CRC. We found that *MTHFR* genotypes 2572C>A, 4869C>G, and 5488C>T were substantially associated with CRC susceptibility and displayed significant combined gene-environment effects (RERI_OR_>0). Moreover, *MTHFR* 2572C>A was associated with increased Hcy and decreased FA levels in the plasma based on *MTHFR* 677CC genotype. To our knowledge, this is the study to provide evidence that 3′-UTR polymorphisms of *MTHFR* gene are associated with CRC susceptibility.

Numerous studies have investigated the associations of *MTHFR* genetic polymorphisms with CRC incidence^8^,^25^. Many reports have demonstrated inconsistent data for the significance of *MTHFR* 677C>T, although the *MTHFR* 677T allele was reported to be a potential genetic risk factor for increased CRC susceptibility, as determined in a recent meta-analysis^25^. There were limited conditions for the association of the *MTHFR* 677T allele with increased CRC risk: (1) high alcohol intake^26^,^27^, (2) low FA intake^26^,^28^, and (3) microsatellite instability (MSI)^29^,^30^,^31^. Although there was no similarity when the present study design was compared with numerous previous reports, the subset-specific associations of 2572C>A, 4869C>G, and 5488C>T polymorphisms were observed. We found common subset-specific associations in the subgroup of patients with the following: ≥62 years of age, male, rectal cancer, tumors ≥5 cm, TNM stage I/II disease, and HTN. We hypothesized the reasons for these correlations were high alcohol intake, low FA intake, and microsatellite instability. Korean men showed higher rates of alcohol consumption than women^32^, FA deficiency increased with age^33^, and FA intake showed stronger associations with decreased risk for the cancer of the rectum than of the colon^34^. HTN may be prevented by FA fortification^35^, and increased prevalence of tumors ≥5 cm were associated with MSI^36^. Finally, CRC with early TNM stage and without lymph node metastases showed a higher frequency of MSI^37^.

The *MTHFR* 677T allele decreased MTHFR activity correlated with increased plasma Hcy and decreased plasma FA levels^13^. Also, we observed that *MTHFR* 2572A allele had a tendency of decreased MTHFR mRNA expression in tumor-adjacent tissues. Therefore, it is necessary to gain an understanding of carcinogenic events caused by decreased MTHFR activity to explain why 3′-UTR polymorphisms may affect CRC susceptibility. Lower MTHFR activity increases Hcy and decrease FA levels in the plasma, inducing development of CRC^38^,^39^. Plasma FA concentration inversely correlates with Hcy level^40^. Depletion of FA may be considered a risk factor for colorectal carcinogenesis because it induces breaks in human chromosomal DNA^41^. Two plausible mechanisms by which FA deficiency may create such breaks are uracil misincorporation and impaired DNA repair^42^,^43^. FA deficiency reduces synthesis of deoxythymidylate from deoxyuridylate, and the resultant nucleotide imbalance accelerates the incorporation of uracil into DNA. Uracil in DNA is excised by a repair glycosylase and, in the process, a transient single-strand break develops in the DNA^44^. Simultaneous removal and repair of two adjacent uracil residues on opposite strands can cause a double-strand break, which is difficult to repair and further increases genetic instability. Unrepaired double-strand DNA breaks enhance cellular transformation and increase cancer risk^44^. FA status is also important to modify cell proliferation rates. James *et al.* reported excessive cell proliferation in livers of FA/methyl-deficient rats^45^. Conversely, FA supplementation has been found to diminish colorectal mucosal proliferation in both animal and human studies. Nensey *et al.* reported the same phenomenon in an animal model^46^. FA supplementation reduces carcinogen-induced ornithine decarboxylase and tyrosine kinase activities, both of which are indicators of cell proliferation^46^. Biasco *et al.*^47^ reported that FA supplementation significantly decreases rectal mucosal proliferation in patients with long-standing ulcerative colitis, a condition that carries a higher risk of CRC, a predisposition to which is considered to be due in part to reduced availability of FA. Akoglu *et al.*^48^ described a human colon cancer cell line in which dihydrofolate and methyl-THF serve as growth-inhibitory factors.

Epidemiologic studies over the past two or three decades have described an inverse relationship between FA status (assessed by dietary FA intake or measurement of red cell and plasma FA levels) with the risk of cancer of the lungs, oropharynx, esophagus, stomach, colorectum, pancreas, cervix, ovary, prostate, and breast, and the risk of neuroblastoma and leukemia^49^,^50^. Although the results of epidemiologic and clinical studies are inconsistent, the reports have indicated 20%–40% lower risk of CRC in subjects with the highest dietary intake or blood levels of folate compared with those with the lowest intake or blood levels^49^,^51^,^52^. Several intervention studies have shown that FA supplementation can improve or reverse poor prognostic factors of CRC^49^,^51^, and some epidemiologic studies have shown a beneficial effect of taking multivitamin supplements containing ≥400 μg FA on CRC risk and mortality^53^,^54^,^55^. The data from animal studies generally support a causal association between FA deficiency and CRC risk and an inhibitory effect of modest levels of FA supplementation on colorectal carcinogenesis^51^. The results for previous association studies of plasma Hcy or FA levels with risk of colorectal neoplasia are complicated. Four studies reported increased risk of colorectal neoplasia by higher plasma Hcy or lower plasma FA levels^56^,^57^,^58^,^59^, Kato *et al.*^56^ and Pufulete *et al.*^57^ demonstrated associations with decreased plasma FA levels whereas Ulvik *et al.*^58^ and Martinez *et al.*^59^ presented significant relationships with higher plasma Hcy levels. Moreover, Martinez *et al.* reported that the risk of colorectal neoplasia could be controlled by FA supplementation^59^. Three other studies did not report a significantly elevated risk of colorectal neoplasia with increasing plasma Hcy or decreasing plasma FA levels^60^,^61^,^62^. In this study, there were the additive interactions of CRC risk (RERI_OR_  >0) between *MTHFR* 3′-UTR minor genotypes and lower plasma FA levels (<5.77 ng/mL: the lowest tertile interval). FA supplementation or fortification may therefore be essential for the prevention of colorectal carcinogenesis in individuals with *MTHFR* 3′-UTR minor genotypes.

Genetic variation in the 3′-UTR region could affect the stability and translation of the mRNA through altered miRNA-binding affinity. In the present study, we could not demonstrate altered miRNA-binding activity depending on *MTHFR* 3′-UTR polymorphisms. One study showed altered miR-149–binding activity with polymorphism *MTHFR* 2572C>A^63^. The *MTHFR* 2572A allele augmented miR-149–binding activity associated with decreased MTHFR expression. Further studies are needed to directly test for binding activity of miRNA to *MTHFR* 3′-UTR polymorphic regions to determine the mechanism by which these polymorphisms may influence cellular proliferation and cancer progression. These studies may have great clinical impact for all diseases related to one-carbon metabolism.

In conclusion, we investigated the relationship of the polymorphisms *MTHFR* 2572C>A, 4869C>G, 5488C>T, and 6685T>C with CRC susceptibility. We found specific 3′-UTR polymorphisms positively correlated with CRC susceptibility, depending on a diversity of clinical and environmental risk factors. This study has several limitations. First, the manner in which 3′-UTR polymorphisms in the *MTHFR* gene affect development of CRC is still unclear. Second, information regarding additional environmental risk factors in CRC patients is lacking and remains to be investigated. Third, a limited number of 3′-UTR polymorphisms were studied. Lastly, the population of this study was limited to ethnic Koreans. Although results of our study provide the first evidence for 3′-UTR variants in the *MTHFR* gene as potential biomarkers for use in CRC prevention, a prospective study on a larger cohort of ethnically diverse patients is warranted to validate these findings.

## Methods

### Ethics statement

The study protocol was approved by the Institutional Review Board of CHA Bundang Medical Center. All study subjects provided written informed consent to participate in the study. All the methods applied in the study were carried out in accordance with the approved guidelines.

### Study population

A case–control study of 850 individuals was conducted. Four hundred and fifty patients diagnosed with CRC at CHA Bundang Medical Center, Seongnam, South Korea were enrolled from June 2005 to January 2010. This study only included CRC patients who had undergone surgical resection with a curative intent and who had histologically confirmed adenocarcinoma. The response rate of CRC patients, with an initial number of 598 individuals, who gave written informed consent was 75.3%. Within the CRC cohort, 155 consecutive patients with proximal colon cancer (i.e., from the cecum to the splenic flexure), 101 consecutive patients with distal colon cancer (i.e., descending and sigmoid colon), 8 consecutive patients with mixed colon cancer, and 186 consecutive patients with rectal cancer underwent primary surgery. Information concerning the date of diagnosis and pathological stage was obtained retrospectively. Tumor staging was performed according to the sixth edition of the American Joint Committee on Cancer (AJCC) staging manual. The control group consisted of 400 individuals randomly selected following a health screening. Of the initial 612 normal visitors, 476 individuals gave written informed consent (response rate: 77.8%), and 76 participants were excluded from the study due to a history of thrombotic diseases or cancer. Patients with a high baseline blood pressure (systolic ≥140 mm Hg or diastolic ≥90 mm Hg) on more than one occasion or a history of treatment with antihypertensive medication were classified as having HTN. Patients with high fasting plasma glucose ( ≥126 mg/dl) and those who took oral hypoglycemic agents or who had a history of insulin treatment were classified as having DM. Individuals were diagnosed with MetS if they possessed three or more of the following five risk factors: BMI ≥25.0 kg/m^2^; TG ≥150 mg/dl; HDL-C <40 mg/dl for men or <50 mg/dl for women; blood pressure ≥130/85 mm Hg or currently taking anti-hypertension medication; and fasting blood sugar ≥100 mg/dl or currently taking hypoglycemic medication.

### Phenotype measurements

Anthropometric measurements included BMI. Systolic and diastolic blood pressures of subjects were measured in the seated position after 10 min of rest. For measurements of physiological parameters, 3-ml samples of blood were obtained after fasting overnight. Plasma glucose was determined in duplicate using the hexokinase method adapted for an automated analyzer (TBA 200FR NEO, Toshiba Medical Systems, Tokyo, Japan). Levels of TG and HDL-C were determined by enzymatic colorimetric methods using commercial reagent sets (Toshiba Medical Systems). The concentration of Hcy in the plasma was measured by fluorescence polarization immunoassay (FPIA) with the IMx automated analyzer (Abbott Laboratories, Chicago, IL, USA). The plasma concentration of FA was determined using a radioassay kit (ACS:180, Bayer, Tarrytown, NY, USA).

### Genotyping

DNA was extracted from leukocytes using a G-DEX™ II Genomic DNA Extraction kit (Intron Biotechnology, Seongnam, Korea) according to the manufacturer’s instructions. Genotyping of the *MTHFR* 677C>T, *MTHFR* 5488C>T, and *MTHFR* 6685T>C was carried out using real-time polymerase chain reaction (PCR) (RG-6000, Corbett Research, Australia) for allelic discrimination, Genotyping of *MTHFR* 2572C>A and *MTHFR* 4869C>G was carried out by PCR-restriction fragment length polymorphism (PCR-RFLP) analysis. Primers and TaqMan probes were designed using Primer Express Software (version 2.0) and were synthesized and supplied by Applied Biosystems (Carlsbad, CA, USA). The reporter dyes used were FAM and JOE. Primers for amplification of *MTHFR* 677C>T were (forward) 5′- TGA CCT GAA GCA CTT GAA GGA GAA -3′ and (reverse) 5′- GGA AGA ATG TGT CAG CCT CAA AGA -3′. Selected probes were 5′-FAM-ATG AAA TCG GCT CCC G -TAMRA-3′ (C-allele–detecting) and 5′-JOE-ATG AAA TCG ACT CCC G -TAMRA-3′ (T-allele–detecting). Primers for amplification of *MTHFR* 5488C>T were (forward) 5′- GAG GCA CCA GCT CTG TGG -3′ and (reverse) 5′- CCC CAG GAA GTC CAA GC -3′. The selected probes were 5′-FAM- CAG CAG CTG CGG GTC TGA A -TAMRA-3′ (C-allele–detecting) and 5′-JOE- CAG CAG CTG TGG GTC TGA A -TAMRA-3′ (T-allele–detecting). Primers for amplification of *MTHFR* 6685T>C were (forward) 5′- CCA GAC CAG AAG CAG TTA -3′ and (reverse) 5′- GCT GTG CAG TGT CAT TT -3′. The selected probes were 5′-FAM- CAC CAA CAA ATG GTG ATA AG -TAMRA-3′ (T-allele–detecting) and 5′-JOE- CAC CAA CAA GTG GTG ATA AG -TAMRA-3′ (C-allele–detecting). *MTHFR* 2572C>A was amplified using forward (5′- TTG CCA ACT AAG CCC TCG AAA CAA -3′) and reverse (5′- TGC CAC ATC TCT TCT ACG ATG CCA -3′) primers. The 140-bp PCR product was then digested with 5U *Sty*I. A digestion product of 140 bp represented the AA genotype; fragments of 140 and 70 bp represented the CA genotype; and 70-bp products represented the CC genotype. *MTHFR* 4869C>G was amplified using forward (5′- AGG CAA GCC CCT CAG CCC TT -3′) and reverse (5′- TCC AGC CCT GAG CCC AGA GTC T -3′) primers. The 126-bp product was digested with 3U *Bsm*AI for 16 h at 55 °C. A restriction fragment of 126 bp represented the CC genotype; fragments of 126 bp, 98 bp, and 28 bp represented the CG genotype; and 98-bp and 28-bp products represented the GG genotype. All genotyping data of study participants were confirmed three times to eliminate genotyping errors. Thirty percent of the PCR assays for each polymorphism were randomly selected and repeated, followed by DNA sequencing, to validate the experimental findings. Sequencing was performed using an ABI 3730xl DNA Analyzer (Applied Biosystems, Carlsbad, CA, USA). The concordance of the quality control samples was 100%.

### Quantitative real-time PCR

To perform quantitative real-time PCR (qRT-PCR), total RNA was extracted from 47 tumor and tumor-adjacent tissues of CRC patients using TRIzol Reagent (Invitrogen, Grand Island, NY, USA) according to the manufacturer’s instructions. cDNA was made from total RNA using the SuperScript III First-Strand Synthesis System (Invitrogen). Quantitation of MTHFR mRNA was determined using real-time PCR (RG-6000, Corbett Research, Australia). The MTHFR mRNA expression ratio (amount of MTHFR mRNA/amount of 18S rRNA) was expressed as –ΔC_T_  = –(C_T MTHFR_ – C_T 18S rRNA_). Housekeeping 18S rRNA was used as an internal control. The primer sequences for amplification were as follows: 18S rRNA, (forward) 5′- AAC TTT CGA TGG TAG TCG CCG -3′ and (reverse) 5′- CCT TGG ATG TGG TAG CCG TTT -3′; MTHFR, (forward) 5′- GAA CGA AGC CAG AGG AAA CA -3′ and (reverse) 5′- GGG TGG AAC ATC TCG AAC TAT C -3′.

### Statistical analysis

To analyze baseline characteristics, chi-square tests were used for categorical data, and Student’s *t*-tests were used for continuous data to compare patient and control baseline data. Association of *MTHFR* 3′-UTR polymorphisms with CRC incidence was calculated using adjusted odds ratios (AORs) and 95% confidence intervals (CIs) from multivariate logistic regression adjusted for age, gender, HTN, DM, BMI, TG, and HDL-C. These parameters were selected as adjustment variables because they were directly or potentially associated with CRC^3^,^18^,^19^,^20^. To evaluate the association data by the Benjamini-Hochberg method, we calculated FDR-corrected *P* values according to the number of genetic markers and stratified groups^64^. RERI_OR_ was used to calculate additive interactions between genotypes and environmental risk factors^24^. When RERI_OR_  >0, there was an additive interaction between combined factors. Correlation of *MTHFR* 3′-UTR polymorphisms with plasma Hcy and FA levels was calculated using regression coefficients and t values from multivariate linear regression adjusted for age, gender, HTN, DM, BMI, TG, and HDL-C. The statistical significances of MTHFR mRNA expression levels according to studied polymorphisms and tissue differences were calculated by Mann-Whitney, Kruskal-Wallis, and Wilcoxon tests. The haplotypes for multiple loci were estimated using the expectation maximization algorithm with SNPAlyze (version 5.1; DYNACOM Co, Ltd, Yokohama, Japan), and those with frequencies of <1% were excluded from statistical analysis. Analyses were performed using GraphPad Prism 4.0 (GraphPad Software Inc., San Diego, CA, USA) and Medcalc version 12.7.1.0 (Medcalc Software, Mariakerke, Belgium).

## Additional Information

**How to cite this article**: Jeon, Y. J. *et al.* Genetic variants in 3'-UTRs of methylenetetrahydrofolate reductase (MTHFR) predict colorectal cancer susceptibility in Koreans. *Sci. Rep.* **5**, 11006; doi: 10.1038/srep11006 (2015).

## Supplementary Material

Supplementary Information

## Figures and Tables

**Table 1:** Baseline characteristics of colorectal cancer patients and control subjects.

**Characteristics**	**Control**	**CRC**	**P**
N	400	450	
Age: years (mean ± SD)	60.89 ± 11.72	62.05 ± 12.29	0.162
Gender male: n (%)	170 (42.5)	212 (47.1)	0.177
Hypertension: n (%)	157 (39.3)	279 (62.0)	<.001
Diabetes mellitus: n (%)	166 (41.5)	253 (56.2)	<.001
BMI ≥ 25 kg/m^2^: n (%)	93 (23.3)	116 (25.8)	0.393
HDL-C <40 (male) or 50 (female) mg/dl: n (%)	78 (19.5)	197 (43.8)	<.001
Triglycerides ≥ 150 mg/dl: n (%)	135 (33.8)	113 (25.1)	0.006
Metabolic syndrome: n (%)	95 (23.8)	171 (38.0)	<.001
Plasma homocysteine levels: μmol/l (n)	9.80 ± 4.17 (395)	10.51 ± 7.76 (383)	0.115
Plasma folate levels: ng/ml (n)	8.85 ± 8.06 (392)	7.77 ± 6.65 (381)	0.043
*MTHFR* 677CC: n (%)	147 (36.8)	165 (36.7)	0.880
*MTHFR* 677CT: n (%)	197 (49.3)	227 (50.4)	
*MTHFR* 677TT: n (%)	56 (14.0)	58 (12.9)	
Tumor site: n (%)			
Colon	—	264 (58.7)	
Rectum	—	186 (41.3)	
Tumor size: n (%)			
<5 cm	—	181 (40.2)	
≥5 cm	—	269 (59.8)	
TNM stage: n (%)			
I	—	42 (9.3)	
II	—	189 (42.0)	
III	—	173 (38.4)	
IV	—	46 (10.2)	

Abbreviations: CRC, colorectal cancer; SD, standard deviation; BMI, body mass index; HDL-C, high density lipoprotein-cholesterol; *MTHFR*, methylenetetrahydrofolate reductase; TNM, tumor node metastasis. *P*-values were calculated by chi-square test for categorical data and two-sided t-test for continuous data.

**Table 2:** Frequencies of *MTHFR* 3′-UTR polymorphisms between CRC patients and control subjects.

**Characteristics**	**Control** (**n = 400)**	**CRC (n = 450)**	**AOR (95% CI)**	**P**	**FDR-P**
*MTHFR* 2572C >A					
CC	278 (69.5)	276 (61.3)	1.00 (ref)		
CA	113 (28.3)	157 (34.9)	1.44 (1.05–1.98)	0.022	0.029
AA	9 (2.3)	17 (3.8)	2.03 (0.85–4.86)	0.110	0.348
Dominant			1.49 (1.10–2.03)	0.010	0.013
Recessive			1.87 (0.77–4.51)	0.166	0.348
HWE *P*	0.529	0.357			
*MTHFR* 4869C >G					
CC	360 (90.0)	365 (81.1)	1.00 (ref)		
CG	40 (10.0)	83 (18.4)	2.12 (1.37–3.26)	<.001	0.002
GG	0 (0.0)	2 (0.4)	NA	>.999	0.999
Dominant			2.17 (1.41–3.33)	<.001	0.001
Recessive			NA	>.999	>.999
HWE *P*	0.293	0.234			
*MTHFR* 5488C >T					
CC	340 (85.0)	352 (78.2)	1.00 (ref)		
CT	59 (14.8)	96 (21.3)	1.64 (1.12–2.41)	0.011	0.022
TT	1 (0.3)	2 (0.4)	2.54 (0.19–34.43)	0.483	0.644
Dominant			1.66 (1.13–2.42)	0.010	0.013
Recessive			2.40 (0.17–33.05)	0.514	0.685
HWE *P*	0.347	0.090			
*MTHFR* 6685T >C					
TT	319 (79.8)	361 (80.2)	1.00 (ref)		
TC	79 (19.8)	82 (18.2)	0.95 (0.65–1.37)	0.767	0.767
CC	2 (0.5)	7 (1.6)	3.12 (0.61–16.07)	0.174	0.348
Dominant			1.00 (0.70–1.44)	0.999	0.999
Recessive			3.13 (0.60–16.24)	0.174	0.348
HWE *P*	0.215	0.352			

Abbreviations: *MTHFR*, methylenetetrahydrofolate reductase; CRC, colorectal cancer; AOR, adjusted odds ratio (adjusted by age, gender, hypertension, diabetes mellitus, body mass index, triglycerides, and high density lipoprotein-cholesterol); CI, confidence interval; FDR, false discovery rate; HWE, Hardy-Weinberg equilibrium; NA, not available.

**Table 3:** Combined genotype and haplotype frequencies of *MTHFR* 3′-UTR polymorphisms between CRC patients and control subjects.

**Characteristics**		**Control (n = 400)**	**CRC (n = 450)**	**AOR(95% CI)**	**P**	**FDR-P**
MTHFR 2572	MTHFR 4869					
CC	CC	278 (69.5)	272 (60.4)	1.00 (ref)		
CC	CG + GG	0 (0.0)	4 (0.9)	NA	>.999	>.999
CA + AA	CC	82 (20.5)	93 (20.7)	1.24 (0.86–1.78)	0.254	0.381
CA + AA	CG + GG	40 (10.0)	81 (18.0)	2.09 (1.35–3.24)	<.001	0.003
MTHFR 2572	MTHFR 5488					
CC	CC	278 (69.5)	266 (59.1)	1.00 (ref)		
CC	CT + TT	0 (0.0)	10 (2.2)	NA	>.999	>.999
CA + AA	CC	62 (15.5)	86 (19.1)	1.53 (1.03–2.26)	0.034	0.051
CA + AA	CT + TT	60 (15.0)	88 (19.6)	1.58 (1.07–2.34)	0.022	0.051
MTHFR 2572	MTHFR 6685					
CC	TT	266 (66.5)	266 (59.1)	1.00 (ref)		
CC	TC + CC	12 (3.0)	10 (2.2)	1.02 (0.42–2.49)	0.963	0.963
CA + AA	TT	53 (13.3)	95 (21.1)	1.89 (1.27–2.82)	0.002	0.006
CA + AA	TC + CC	69 (17.3)	79 (17.6)	1.18 (0.80–1.74)	0.415	0.623
MTHFR 4869	MTHFR 5488					
CC	CC	340 (85.0)	352 (78.2)	1.00 (ref)		
CC	CT + TT	20 (5.0)	13 (2.9)	0.69 (0.32–1.48)	0.339	0.339
CG + GG	CC	0 (0.0)	0 (0.0)	NA	NA	NA
CG + GG	CT + TT	40 (10.0)	85 (18.9)	2.12 (1.38–3.27)	<.001	0.001
MTHFR 4869	MTHFR 6685					
CC	TT	287 (71.8)	286 (63.6)	1.00 (ref)		
CC	TC + CC	73 (18.3)	79 (17.6)	1.14 (0.78–1.68)	0.503	0.674
CG + GG	TT	32 (8.0)	75 (16.7)	2.41 (1.51–3.86)	<.001	<.001
CG + GG	TC + CC	8 (2.0)	10 (2.2)	1.24 (0.45–3.43)	0.674	0.674
MTHFR 5488	MTHFR 6685					
CC	TT	273 (68.3)	277 (61.6)	1.00 (ref)		
CC	TC + CC	67 (16.8)	75 (16.7)	1.14 (0.77–1.69)	0.511	0.767
CT + TT	TT	46 (11.5)	84 (18.7)	1.85 (1.22–2.82)	0.004	0.012
CT + TT	TC + CC	14 (3.5)	14 (3.1)	1.03 (0.46–2.34)	0.938	0.938
MTHFR 2572/4869/5488/6685 haplotypes						
2572C-4869C-5488C-6685T		636 (79.5)	675 (75.0)	1.00 (ref)		
2572A-4869C-5488C-6685C		66 (8.3)	78 (8.7)	1.12 (0.77–1.62)	0.557	0.557
2572A-4869G-5488T-6685T		40 (5.0)	83 (9.2)	1.99 (1.31–3.00)	0.001	0.002

Abbreviations: *MTHFR*, methylenetetrahydrofolate reductase; CRC, colorectal cancer; AOR, adjusted odds ratio (adjusted by age, gender, hypertension, diabetes mellitus, body mass index, triglycerides, and high density lipoprotein-cholesterol); CI, confidence interval; FDR, false discovery rate; NA, not available. Haplotypes of frequencies <1% were excluded from the analysis.

**Table 4:** Stratified effects of *MTHFR* 2572C>A, 4869C>G, 5488C>T, and 6685T>C on CRC susceptibility.

	**MTHFR 2572CA + AA**		**MTHFR 4869CG + GG**		**MTHFR 5488CT + TT**		**MTHFR 6685TC + CC**	
**Variables**	**AOR (95% CI)**	**FDR-P**	**AOR (95% CI)**	**FDR-P**	**AOR (95% CI)**	**FDR-P**	**AOR (95% CI)**	**FDR-P**
Age								
<62 years	1.36 (0.89–2.10)	0.219	2.09 (1.15–3.78)	0.044	1.44 (0.86–2.41)	0.219	1.06 (0.62–1.83)	0.843
≥62 years	1.72 (1.10–2.69)	0.044	2.35 (1.24–4.45)	0.044	1.96 (1.10–3.49)	0.044	1.05 (0.63–1.76)	0.843
Gender								
Male	2.52 (1.57–4.04)	<.001	5.05 (2.35–10.87)	<.001	2.99 (1.59–5.63)	0.002	1.16 (0.69–1.97)	0.809
Female	1.04 (0.68–1.57)	0.866	1.32 (0.76–2.28)	0.656	1.14 (0.69–1.87)	0.809	0.95 (0.56–1.62)	0.856
Tumor site								
Colon	1.40 (0.98–1.99)	0.100	1.77 (1.09–2.90)	0.044	1.49 (0.96–2.31)	0.100	0.97 (0.64–1.49)	0.904
Rectum	1.89 (1.27–2.80)	0.008	2.70 (1.59–4.59)	0.002	2.02 (1.25–3.27)	0.011	1.18 (0.74–1.87)	0.562
Tumor size								
<5 cm	1.30 (0.87–1.93)	0.315	1.64 (0.94–2.86)	0.158	1.33 (0.81–2.18)	0.347	0.83 (0.51–1.36)	0.459
≥5 cm	1.75 (1.23–2.48)	0.005	2.64 (1.64–4.24)	<.001	2.02 (1.31–3.10)	0.004	1.20 (0.79–1.82)	0.440
TNM stage								
I + II	1.70 (1.19–2.45)	0.016	2.33 (1.42–3.83)	0.007	1.84 (1.18–2.87)	0.018	1.18 (0.77–1.81)	0.520
III + IV	1.41 (0.97–2.05)	0.097	1.99 (1.19–3.32)	0.018	1.54 (0.97–2.45)	0.097	0.94 (0.59–1.49)	0.788
MetS								
No	1.39 (0.96–2.02)	0.108	2.00 (1.19–3.34)	0.056	1.63 (1.02–2.59)	0.080	0.92 (0.59–1.43)	0.703
Yes	1.99 (1.11–3.56)	0.056	2.88 (1.22–6.80)	0.056	1.96 (0.96–4.01)	0.102	1.33 (0.67–2.62)	0.473
HTN								
No	1.11 (0.72–1.72)	0.640	2.02 (1.11–3.69)	0.044	1.17 (0.68–1.99)	0.640	0.88 (0.52–1.49)	0.640
Yes	2.03 (1.30–3.17)	0.012	2.32 (1.24–4.35)	0.012	2.42 (1.35–4.34)	0.012	1.21 (0.71–2.06)	0.640
DM								
No	1.18 (0.77–1.83)	0.513	1.89 (1.01–3.54)	0.092	1.47 (0.84–2.58)	0.278	0.86 (0.51–1.44)	0.563
Yes	2.03 (1.30–3.17)	0.012	2.55 (1.38–4.70)	0.012	1.88 (1.11–3.20)	0.053	1.31 (0.77–2.26)	0.429
BMI								
<25 kg/m^2^	1.47 (1.03–2.10)	0.088	2.12 (1.31–3.44)	0.016	1.75 (1.13–2.71)	0.048	0.93 (0.60–1.44)	0.760
≥25 kg/m^2^	1.59 (0.86–2.95)	0.224	2.43 (0.92–6.40)	0.146	1.36 (0.63–2.97)	0.523	1.30 (0.65–2.60)	0.523
TG								
<150 mg/dl	1.30 (0.90–1.89)	0.212	1.68 (1.02–2.79)	0.086	1.61 (1.01–2.54)	0.086	0.99 (0.63–1.55)	0.967
≥150 mg/dl	2.20 (1.22–3.97)	0.036	4.20 (1.73–10.22)	0.016	1.80 (0.87–3.73)	0.178	1.04 (0.53–2.03)	0.967
HDL-C								
<40 (M)/50 (F) mg/dl	1.90 (1.02–3.54)	0.115	1.23 (0.54–2.79)	0.698	1.16 (0.55–2.43)	0.698	1.51 (0.72–3.18)	0.448
≥40 (M)/50 (F) mg/dl	1.38 (0.97–1.98)	0.152	2.59 (1.58–4.26)	0.002	1.86 (1.20–2.88)	0.024	0.88 (0.57–1.36)	0.698

Abbreviations: *MTHFR*, methylenetetrahydrofolate reductase; CRC, colorectal cancer; AOR, adjusted odds ratio(adjusted by age, gender, HTN, DM, BMI, TG, and HDL-C); CI, confidence interval; FDR, false discovery rate; TNM, tumor node metastasis; MetS, metabolic syndrome; HTN, hypertension; DM, diabetes mellitus; BMI, body mass index; TG, triglycerides; HDL-C, high density lipoprotein-cholesterol; M, male; F, female.

**Table 5:** Combined effects between *MTHFR* 3′-UTR polymorphisms and plasma folate levels on CRC risk.

	** ≥5.77 ng/mL of folate**		<**5.77 ng/mL of folate**		
	**AOR(95% CI)**	***P***	**AOR(95% CI)**	***P***	**RERI_OR_(95% CI)**
*MTHFR* 2572CC	1.00 (ref)		1.49 (0.95–2.32)	0.079	
*MTHFR* 2572CA + AA	1.13 (0.72–1.75)	0.597	3.74 (2.04–6.87)	<0.001	2.12 (1.37–3.80)
*MTHFR* 4869CC	1.00 (ref)		1.84 (1.25–2.73)	0.002	
*MTHFR* 4869CG + GG	1.97 (1.08–3.59)	0.027	4.41 (1.71–11.35)	0.002	1.60 (0.38–6.03)
*MTHFR* 5488CC	1.00 (ref)		1.72 (1.16–2.54)	0.007	
*MTHFR* 5488CT + TT	1.34 (0.79–2.27)	0.286	4.40 (1.74–11.11)	0.002	2.34 (0.79–7.30)
*MTHFR* 6685TT	1.00 (ref)		1.44 (0.97–2.16)	0.074	
*MTHFR* 6685TC + CC	0.67 (0.38–1.17)	0.160	3.31 (1.60–6.86)	0.001	2.20 (1.25–4.53)

Abbreviations: *MTHFR*, methylenetetrahydrofolate reductase; CRC, colorectal cancer; AOR, adjusted odds ratio (adjusted by age, gender, hypertension, diabetes mellitus, body mass index, triglycerides, and high density lipoprotein-cholesterol); CI, confidence interval; RERI_OR_, relative excess odds due to interaction. <5.77 ng/mL of folate was the lowest tertile interval.

**Table 6:** Multiple linear regression analysis of plasma homocysteine and folate levels according to *MTHFR* 3′-UTR polymorphisms.

		**Homocysteine**			**Folate**		
**Filter condition**	**Polymorphism**	**Coefficient**	**t value**	**P**	**Coefficient**	**t value**	**P**
None	*MTHFR* 677C >T	1.172	3.083	0.002	–0.708	–2.320	0.021
None	*MTHFR* 2572C >A	–0.439	–0.989	0.323	–0.034	–0.097	0.923
None	*MTHFR* 4869C >G	–1.071	–1.582	0.114	0.381	0.699	0.485
None	*MTHFR* 5488C >T	–0.608	–1.030	0.304	0.636	1.348	0.178
None	*MTHFR* 6685T >C	–0.027	–0.049	0.961	–0.693	–1.541	0.124
*MTHFR* 677CC	*MTHFR* 2572C >A	1.120	3.361	0.001	–1.370	–2.595	0.010
*MTHFR* 677CC	*MTHFR* 4869C >G	0.019	0.041	0.968	–0.509	–0.669	0.504
*MTHFR* 677CC	*MTHFR* 5488C >T	0.777	1.911	0.057	–0.546	–0.851	0.396
*MTHFR* 677CC	*MTHFR* 6685T >C	0.706	1.802	0.073	–1.482	–2.428	0.016
*MTHFR* 677CT	*MTHFR* 2572C >A	–0.926	–1.129	0.260	0.552	1.040	0.299
*MTHFR* 677CT	*MTHFR* 4869C >G	–1.313	–1.080	0.281	0.674	0.855	0.393
*MTHFR* 677CT	*MTHFR* 5488C >T	–1.290	–1.170	0.243	1.393	1.958	0.051
*MTHFR* 677CT	*MTHFR* 6685T >C	0.229	0.216	0.830	–0.529	–0.770	0.442
*MTHFR* 677TT	*MTHFR* 2572C >A	0.956	0.306	0.761	–3.115	–0.837	0.406
*MTHFR* 677TT	*MTHFR* 4869C >G	NA	NA	NA	NA	NA	NA
*MTHFR* 677TT	*MTHFR* 5488C >T	NA	NA	NA	NA	NA	NA
*MTHFR* 677TT	*MTHFR* 6685T >C	NA	NA	NA	NA	NA	NA

Abbreviations: *MTHFR*, methylenetetrahydrofolate reductase; NA, not available. Regression coefficients, t values, and *P* values were calculated by multiple linear regression analysis including age, gender, hypertension, diabetes mellitus, body mass index, triglycerides, and high density lipoprotein-cholesterol.
