# Supplementary material for: Genetic variants in 3′-UTRs of methylenetetrahydrofolate reductase (MTHFR) predict colorectal cancer susceptibility in Koreans
Source: Sci Rep. 2015 Jun 5;5:11006. doi: 10.1038/srep11006 (PMC4457011; doi:10.1038/srep11006)
Supplement: Supplementary Information [file srep11006-s1.doc]

**Supplementary Information**

Title: Genetic variants in 3’-UTRs of methylenetetrahydrofolate reductase (*MTHFR*) predict colorectal cancer susceptibility in Koreans

Young Joo Jeon^1,*^, Jong Woo Kim^2,*^, Hye Mi Park^1^, Jung O Kim^1^, Hyo Geun Jang^1^, Jisu Oh^3^, Seong Gyu Hwang^3^, Sung Won Kwon^2^, Doyeun Oh^3^, Nam Keun Kim^1^

^1^Institute for Clinical Research, School of Medicine, CHA University, 351 Yatap-dong, Bundang-gu, Seongnam 463-712, South Korea, ^2^Department of Surgery, School of Medicine, CHA University, 351 Yatap-dong, Bundang-gu, Seongnam 463-712, South Korea, ^3^Department of Internal Medicine, School of Medicine, CHA University, 351 Yatap-dong, Bundang-gu, Seongnam 463-712, South Korea

^*^ Young Joo Jeon and Jong Woo Kim contributed equally to this work.

| **Supplementary Table S1** Minor allele frequencies of *MTHFR* 3’-UTR polymorphisms in previous reports | | | | | | | | | | | |
| --- | --- | --- | --- | --- | --- | --- | --- | --- | --- | --- | --- |
|  |  |  |  | *MTHFR* 2572C>A | | *MTHFR* 4869C>G | | *MTHFR* 5488C>T | | *MTHFR* 6685T>C | |
| Study | Reference | N | Race | C allele | A allele | C allele | G allele | C allele | T allele | T allele | C allele |
| Spellicy et al. | 1 | 120 | White | 0.78 | 0.22 | - | - | - | - | - | - |
| Spellicy et al. | 1 | 114 | Hispanic | 0.81 | 0.19 | - | - | - | - | - | - |
| Liu et al. | 2 | 515 | American | 0.70 | 0.30 | - | - | - | - | 0.68 | 0.32 |
| Wu et al. | 3 | 444 | Chinese | 0.80 | 0.20 | - | - | - | - | - | - |
| Deroo et al. | 4 | 717 | American | - | - | 0.90 | 0.10 | 0.96 | 0.04 | - | - |
| Liu et al. | 5 | 1873 | American | 0.67 | 0.33 | - | - | - | - | 0.71 | 0.29 |
| Liu et al. | 6 | 517 | Chinese | - | - | - | - | - | - | 0.91 | 0.09 |
| Jeon et al. | (present study) | 400 | Korean | 0.84 | 0.16 | 0.95 | 0.05 | 0.92 | 0.08 | 0.90 | 0.10 |

**References**

1. Spellicy, C. J. *et al*. Folate metabolism gene 5,10-methylenetetrahydrofolate reductase (MTHFR) is associated with ADHD in myelomeningocele patients. *PLoS One* **7**, e51330 (2012).
2. Liu, P., Lu, Y., Recker, R. R., Deng, H. W. & Dvornyk, V. Association analyses suggest multiple interaction effects of the methylenetetrahydrofolate reductase polymorphisms on timing of menarche and natural menopause in white women. *Menopause* **17**, 185–190 (2010).
3. Wu, C. *et al*. The human MTHFR rs4846049 polymorphism increases coronary heart disease risk through modifying miRNA binding. *Nutr Metab Cardiovasc Dis* **23**, 693–698 (2013).
4. Deroo, L. A. *et al*. Global DNA methylation and one-carbon metabolism gene polymorphisms and the risk of breast cancer in the Sister Study. *Carcinogenesis* **35**, 333–338 (2014).
5. Liu, X. *et al*. The MTHFR gene polymorphism is associated with lean body mass but not fat body mass. *Hum Genet* **123**, 189–196 (2008).
6. Liu, H. *et al*. Association of polymorphisms in one-carbon metabolizing genes and lung cancer risk: a case-control study in Chinese population. *Lung Cancer* **61**, 21–29 (2008).

| **Supplementary Table S2** AOR values of *MTHFR* 3'-UTR genotypes according to confounding variables | | | | | | | | |
| --- | --- | --- | --- | --- | --- | --- | --- | --- |
|  | *MTHFR* 2572CA+AA | | *MTHFR* 4869CG+GG | | *MTHFR* 5488CT+TT | | *MTHFR* 6685TC+CC | |
| Variable | AOR (95% CI) | *P* | AOR (95% CI) | *P* | AOR (95% CI) | *P* | AOR (95% CI) | *P* |
| None | 1.44 (1.08–1.91) | 0.013 | 2.10 (1.40–3.14) | <.001 | 1.58 (1.11–2.25) | 0.012 | 0.97 (0.69–1.36) | 0.864 |
| Age | 1.44 (1.08–1.92) | 0.012 | 2.11 (1.41–3.16) | <.001 | 1.60 (1.12–2.29) | 0.009 | 0.96 (0.68–1.34) | 0.806 |
| Gender | 1.43 (1.07–1.90) | 0.014 | 2.10 (1.41–3.15) | <.001 | 1.59 (1.11–2.26) | 0.011 | 0.96 (0.69–1.35) | 0.816 |
| HTN | 1.43 (1.07–1.92) | 0.016 | 2.05 (1.36–3.09) | <.001 | 1.58 (1.10–2.27) | 0.013 | 0.99 (0.70–1.40) | 0.962 |
| DM | 1.41 (1.06–1.88) | 0.020 | 2.00 (1.33–3.00) | <.001 | 1.51 (1.05–2.16) | 0.025 | 0.99 (0.70–1.39) | 0.939 |
| BMI | 1.43 (1.08–1.90) | 0.014 | 2.11 (1.41–3.16) | <.001 | 1.58 (1.11–2.25) | 0.011 | 0.96 (0.69–1.35) | 0.817 |
| TG | 1.45 (1.09–1.94) | 0.011 | 2.08 (1.39–3.12) | <.001 | 1.57 (1.10–2.25) | 0.013 | 0.98 (0.70–1.38) | 0.926 |
| HDL-C | 1.52 (1.13–2.04) | 0.006 | 2.24 (1.48–3.39) | <.001 | 1.68 (1.16–2.42) | 0.006 | 0.97 (0.68–1.37) | 0.859 |
| Abbreviations: AOR, adjusted odds ratio; CI, confidence interval; *MTHFR*, methylenetetrahydrofolate reductase; HTN, hypertension; DM, diabetes mellitus; BMI, body mass index; TG, triglycerides; HDL-C, high density lipoprotein-cholesterol. | | | | | | | | |

| **Supplementary Table S3** *MTHFR* 3'-UTR genotypes according to stratified groups of CRC | | | | | | | | |
| --- | --- | --- | --- | --- | --- | --- | --- | --- |
|  | 2572CC/CA/AA | | 4869CC/CG/GG | | 5488CC/CT/TT | | 6685TT/TC/CC | |
| Variables | Control | CRC | Control | CRC | Control | CRC | Control | CRC |
| Age |  |  |  |  |  |  |  |  |
| <62 years | 131/57/6 | 124/71/5 | 173/21/0 | 161/39/0 | 160/33/1 | 155/45/0 | 158/35/1 | 164/33/3 |
| ≥62 years | 147/56/3 | 152/86/12 | 187/19/0 | 204/44/2 | 180/26/0 | 197/51/2 | 161/44/1 | 197/49/4 |
| Gender |  |  |  |  |  |  |  |  |
| Male | 123/43/4 | 119/86/7 | 160/10/0 | 167/43/2 | 152/18/0 | 162/48/2 | 132/37/1 | 166/45/1 |
| Female | 155/70/5 | 157/71/10 | 200/30/0 | 198/40/0 | 188/41/1 | 190/48/0 | 187/42/1 | 195/37/6 |
| Tumor site |  |  |  |  |  |  |  |  |
| Colon | 278/113/9 | 168/88/8 | 360/40/0 | 218/45/1 | 340/59/1 | 210/53/1 | 319/79/2 | 215/46/3 |
| Rectum | 278/113/9 | 108/69/9 | 360/40/0 | 147/38/1 | 340/59/1 | 142/43/1 | 319/79/2 | 146/36/4 |
| Tumor size |  |  |  |  |  |  |  |  |
| <5 cm | 278/113/9 | 118/59/4 | 360/40/0 | 153/27/1 | 340/59/1 | 147/33/1 | 319/79/2 | 152/28/1 |
| ≥5 cm | 278/113/9 | 158/98/13 | 360/40/0 | 212/56/1 | 340/59/1 | 205/63/1 | 319/79/2 | 209/54/6 |
| TNM stage |  |  |  |  |  |  |  |  |
| I+II | 278/113/9 | 137/84/10 | 360/40/0 | 186/44/1 | 340/59/1 | 178/52/1 | 319/79/2 | 182/44/5 |
| III+IV | 278/113/9 | 139/73/7 | 360/40/0 | 179/39/1 | 340/59/1 | 174/44/1 | 319/79/2 | 179/38/2 |
| *MTHFR* 677C>T |  |  |  |  |  |  |  |  |
| 677CC | 74/64/9 | 76/73/16 | 122/25/0 | 118/45/2 | 109/37/1 | 112/51/2 | 96/49/2 | 113/46/6 |
| 677CT | 149/48/0 | 145/82/0 | 182/15/0 | 189/38/0 | 175/22/0 | 182/45/0 | 167/30/0 | 190/36/1 |
| 677TT | 55/1/0 | 55/2/1 | 56/0/0 | 58/0/0 | 56/0/0 | 58/0/0 | 56/0/0 | 58/0/0 |
| MetS |  |  |  |  |  |  |  |  |
| No | 212/85/8 | 178/92/9 | 273/32/0 | 231/47/1 | 260/44/1 | 223/55/1 | 242/61/2 | 228/48/3 |
| Yes | 66/28/1 | 98/65/8 | 87/8/0 | 134/36/1 | 80/15/0 | 129/41/1 | 77/18/0 | 133/34/4 |
| HTN |  |  |  |  |  |  |  |  |
| No | 162/74/7 | 112/50/9 | 218/25/0 | 142/28/1 | 201/41/1 | 139/31/1 | 189/52/2 | 139/29/3 |
| Yes | 116/39/2 | 164/107/8 | 142/15/0 | 223/55/1 | 139/18/0 | 213/65/1 | 130/27/0 | 222/53/4 |
| DM |  |  |  |  |  |  |  |  |
| No | 158/72/4 | 132/59/6 | 211/23/0 | 168/29/0 | 200/33/1 | 163/34/0 | 181/52/1 | 160/35/2 |
| Yes | 120/41/5 | 144/98/11 | 149/17/0 | 197/54/2 | 140/26/0 | 189/62/2 | 138/27/1 | 201/47/5 |
| BMI |  |  |  |  |  |  |  |  |
| <25 kg/m^2^ | 215/84/8 | 209/116/9 | 274/33/0 | 269/64/1 | 261/45/1 | 260/73/1 | 248/57/2 | 275/56/3 |
| ≥25 kg/m^2^ | 63/29/1 | 67/41/8 | 86/7/0 | 96/19/1 | 79/14/0 | 92/23/1 | 71/22/0 | 86/26/4 |
| TG |  |  |  |  |  |  |  |  |
| <150 mg/dl | 184/74/7 | 212/112/13 | 234/31/0 | 276/59/2 | 225/39/1 | 263/72/2 | 215/48/2 | 271/59/7 |
| ≥150 mg/dl | 94/39/2 | 64/45/4 | 126/9/0 | 89/24/0 | 115/20/0 | 89/24/0 | 104/31/0 | 90/23/0 |
| HDL-C |  |  |  |  |  |  |  |  |
| <40(M)/50(F) mg/dl | 60/16/2 | 124/64/9 | 69/9/0 | 167/29/1 | 66/12/0 | 161/35/1 | 66/12/0 | 154/38/5 |
| ≥40(M)/50(F) mg/dl | 218/97/7 | 152/93/8 | 291/31/0 | 198/54/1 | 274/47/1 | 191/61/1 | 253/67/2 | 207/44/2 |
| FA |  |  |  |  |  |  |  |  |
| <5.77 ng/ml | 61/22/1 | 74/53/5 | 77/7/0 | 108/23/1 | 77/7/0 | 104/27/1 | 70/14/0 | 97/32/3 |
| ≥5.77 ng/ml | 165/77/3 | 121/62/4 | 219/26/0 | 151/35/1 | 202/42/1 | 148/38/1 | 195/49/1 | 159/27/1 |
| Abbreviations: TNM, tumor node metastasis; *MTHFR*, methylenetetrahydrofolate reductase; MetS, metabolic syndrome; HTN, hypertension; DM, diabetes mellitus; BMI, body mass index; TG, triglycerides; HDL-C, high density lipoprotein-cholesterol; M, male; F, female; FA, folate. | | | | | | | | |

| **Supplementary Table S4** Combined effects between *MTHFR* 3'-UTR polymorphisms and MetS on CRC risk | | | | | |
| --- | --- | --- | --- | --- | --- |
|  | Without MetS | | With MetS | |  |
|  | AOR (95% CI) | *P* | AOR (95% CI) | *P* | RERI_OR_ (95% CI) |
| *MTHFR* 2572CC | 1.00 (ref) |  | 1.78 (1.22–2.59) | 0.003 |  |
| *MTHFR* 2572CA+AA | 1.39 (0.96–2.02) | 0.081 | 3.03 (1.88–4.89) | <.001 | 0.86 (0.70–1.28) |
| *MTHFR* 4869CC | 1.00 (ref) |  | 1.79 (1.29–2.47) | 0.001 |  |
| *MTHFR* 4869CG+GG | 2.00 (1.19–3.34) | 0.008 | 5.66 (2.57–12.47) | <.001 | 2.87 (1.09–7.66) |
| *MTHFR* 5488CC | 1.00 (ref) |  | 1.86 (1.33–2.60) | <.001 |  |
| *MTHFR* 5488CT+TT | 1.63 (1.02–2.59) | 0.040 | 3.31 (1.78–6.15) | <.001 | 0.82 (0.43–1.96) |
| *MTHFR* 6685TT | 1.00 (ref) |  | 1.83 (1.31–2.57) | 0.001 |  |
| *MTHFR* 6685TC+CC | 0.92 (0.59–1.43) | 0.703 | 2.31 (1.27–4.19) | 0.006 | 0.56 (0.37–1.19) |
| Abbreviations: *MTHFR*, methylenetetrahydrofolate reductase; MetS, metabolic syndrome; CRC, colorectal cancer; AOR, adjusted odds ratio (adjusted by age, gender, hypertension, diabetes mellitus, body mass index, triglycerides, and high density lipoprotein-cholesterol); CI, confidence interval; RERI_OR_, relative excess odds due to interaction. | | | | | |

| **Supplementary Table S5** Combined effects between *MTHFR* 3'-UTR polymorphisms and HTN on CRC risk | | | | | |
| --- | --- | --- | --- | --- | --- |
|  | Without HTN | | With HTN | |  |
|  | AOR (95% CI) | *P* | AOR (95% CI) | *P* | RERI_OR_ (95% CI) |
| *MTHFR* 2572CC | 1.00 (ref) |  | 2.25 (1.53–3.29) | <.001 |  |
| *MTHFR* 2572CA+AA | 1.11 (0.72–1.72) | 0.640 | 5.34 (3.25–8.80) | <.001 | 2.98 (2.00–4.79) |
| *MTHFR* 4869CC | 1.00 (ref) |  | 2.55 (1.82–3.57) | <.001 |  |
| *MTHFR* 4869CG+GG | 2.02 (1.11–3.69) | 0.022 | 6.85 (3.51–13.38) | <.001 | 3.28 (1.58–7.12) |
| *MTHFR* 5488CC | 1.00 (ref) |  | 2.31 (1.64–3.25) | <.001 |  |
| *MTHFR* 5488CT+TT | 1.17 (0.68–1.99) | 0.562 | 6.29 (3.37–11.74) | <.001 | 3.81 (2.05–7.50) |
| *MTHFR* 6685TT | 1.00 (ref) |  | 2.44 (1.73–3.46) | <.001 |  |
| *MTHFR* 6685TC+CC | 0.81 (0.48–1.36) | 0.425 | 3.39 (1.93–5.95) | <.001 | 1.14 (0.72–2.13) |
| Abbreviations: *MTHFR*, methylenetetrahydrofolate reductase; HTN, hypertension; CRC, colorectal cancer; AOR, adjusted odds ratio (adjusted by age, gender, diabetes mellitus, body mass index, triglycerides, and high density lipoprotein-cholesterol); CI, confidence interval; RERI_OR_, relative excess odds due to interaction. | | | | | |

| **Supplementary Table S6** Combined effects between *MTHFR* 3'-UTR polymorphisms and DM on CRC risk | | | | | |
| --- | --- | --- | --- | --- | --- |
|  | Without DM | | With DM | |  |
|  | AOR (95% CI) | *P* | AOR (95% CI) | *P* | RERI_OR_ (95% CI) |
| *MTHFR* 2572CC | 1.00 (ref) |  | 1.11 (0.77–1.60) | 0.576 |  |
| *MTHFR* 2572CA+AA | 1.18 (0.77–1.83) | 0.460 | 2.14 (1.37–3.33) | <.001 | 0.85 (0.83–0.90) |
| *MTHFR* 4869CC | 1.00 (ref) |  | 1.19 (0.86–1.64) | 0.288 |  |
| *MTHFR* 4869CG+GG | 1.89 (1.01–3.54) | 0.047 | 3.03 (1.63–5.61) | <.001 | 0.95 (0.76–1.43) |
| *MTHFR* 5488CC | 1.00 (ref) |  | 1.20 (0.87–1.68) | 0.288 |  |
| *MTHFR* 5488CT+TT | 1.47 (0.84–2.58) | 0.179 | 2.22 (1.29–3.79) | 0.003 | 0.55 (0.53–0.58) |
| *MTHFR* 6685TT | 1.00 (ref) |  | 1.25 (0.90–1.73) | 0.178 |  |
| *MTHFR* 6685TC+CC | 0.86 (0.52–1.44) | 0.566 | 1.46 (0.84–2.52) | 0.174 | <0 |
| Abbreviations: *MTHFR*, methylenetetrahydrofolate reductase; DM, diabetes mellitus; CRC, colorectal cancer; AOR, adjusted odds ratio (adjusted by age, gender, hypertension, body mass index, triglycerides, and high density lipoprotein-cholesterol); CI, confidence interval; RERI_OR_, relative excess odds due to interaction. | | | | | |

| **Supplementary Table S7** Combined effects between *MTHFR* 3'-UTR polymorphisms and HDL-C levels on CRC risk | | | | | |
| --- | --- | --- | --- | --- | --- |
|  | ≥40(M)/50(F) mg/dL of HDL-C | | <40(M)/50(F) mg/dL of HDL-C | |  |
|  | AOR (95% CI) | *P* | AOR (95% CI) | *P* | RERI_OR_ (95% CI) |
| *MTHFR* 2572CC | 1.00 (ref) |  | 2.96 (2.01–4.34) | <.001 |  |
| *MTHFR* 2572CA+AA | 1.38 (0.97–1.98) | 0.080 | 5.62 (3.14–10.06) | <.001 | 2.28 (1.16–4.74) |
| *MTHFR* 4869CC | 1.00 (ref) |  | 3.55 (2.50–5.04) | <.001 |  |
| *MTHFR* 4869CG+GG | 2.59 (1.58–4.26) | <.001 | 4.07 (1.83–9.04) | <.001 | <0 |
| *MTHFR* 5488CC | 1.00 (ref) |  | 3.46 (2.42–4.95) | <.001 |  |
| *MTHFR* 5488CT+TT | 1.86 (1.20–2.88) | 0.005 | 3.72 (1.83–7.56) | <.001 | <0 |
| *MTHFR* 6685TT | 1.00 (ref) |  | 2.80 (1.96–4.00) | <.001 |  |
| *MTHFR* 6685TC+CC | 0.86 (0.55–1.32) | 0.490 | 4.46 (2.23–8.90) | <.001 | 1.80 (0.72–4.58) |
| Abbreviations: *MTHFR*, methylenetetrahydrofolate reductase; HDL-C, high density lipoprotein-cholesterol; M, male; F, female; CRC, colorectal cancer; AOR, adjusted odds ratio (adjusted by age, gender, hypertension, body mass index, and triglycerides); CI, confidence interval; RERI_OR_, relative excess odds due to interaction. | | | | | |

| **Supplementary Table S8** Characteristics between tumor and tumor-adjacent tissues | | | |
| --- | --- | --- | --- |
|  | Adjacent tumor (n=47) | Tumor (n=47) | *P* |
| MTHFR expression (mean±SD) | –7.38±3.55 | –7.81±2.38 | 0.076 |
| Gender (male), n (%) | 28 (59.6) | 28 (59.6) |  |
| TNM stage, n (%) |  |  |  |
| I | 3 (6.4) | 3 (6.4) |  |
| II | 20 (42.6) | 20 (42.6) |  |
| III | 23 (48.9) | 23 (48.9) |  |
| IV | 1 (2.1) | 1 (2.1) |  |
| Abbreviations: MTHFR, methylenetetrahydrofolate reductase; SD, standard deviation; TNM, tumor node metastasis. MTHFR mRNA expression ratios were derived from –ΔC_T_ = – (C_T MTHFR_ – C_T 18S rRNA_). *P*-value for MTHFR expression was calculated by the Mann-Whitney test. | | | |

| **Supplementary Table S9** MTHFR mRNA expression ratios according to *MTHFR* genotypes | | | |
| --- | --- | --- | --- |
|  | Adjacent tumor (n=47) | Tumor (n=47) | *P* |
| MTHFR mRNA expression |  |  |  |
| *MTHFR* 677CC (n=17) | –6.78±2.04 | –7.22±0.60 | 0.119 |
| *MTHFR* 677CT+TT (n=30) | –7.72±4.16 | –8.15±2.91 | 0.266 |
| *P* | 0.400 | 0.319 |  |
| *MTHFR* 2572CC (n=32) | –6.43±2.26 | –7.47±1.24 | 0.012 |
| *MTHFR* 2572CA+AA (n=15) | –9.49±4.90 | –8.51±3.74 | 0.827 |
| *P* | 0.023 | 0.766 |  |
| *MTHFR* 4869CC (n=42) | –7.11±3.26 | –7.53±1.25 | 0.073 |
| *MTHFR* 4869CG+GG (n=5) | –10.15±5.70 | –10.30±6.70 | 1.000 |
| *P* | 0.349 | 0.729 |  |
| *MTHFR* 5488CC (n=40) | –7.11±3.34 | –7.45±1.16 | 0.080 |
| *MTHFR* 5488CT+TT (n=7) | –9.18±4.71 | –9.82±5.37 | 0.699 |
| *P* | 0.359 | 0.613 |  |
| *MTHFR* 6685TT (n=40) | –6.90±2.86 | –7.92±2.56 | 0.018 |
| *MTHFR* 6685TC+CC (n=7) | –10.00±5.71 | –7.22±0.82 | 0.366 |
| *P* | 0.088 | 0.167 |  |
| Abbreviations: MTHFR, methylenetetrahydrofolate reductase. Data were presented as mean ± standard deviation. MTHFR mRNA expression ratios were derived from –ΔC_T_ = – (C_T MTHFR_ – C_T 18S rRNA_). *P*-values were calculated by the Mann-Whitney test. | | | |

| **Supplementary Table S10** MTHFR mRNA expression ratios according to *MTHFR* 677C>T and 2572C>A | | | | |
| --- | --- | --- | --- | --- |
|  | *MTHFR* 677C>T | Adjacent tumor (n=47) | Tumor (n=47) | *P* |
| MTHFR mRNA expression |  |  |  |  |
| *MTHFR* 2572CC (n=7) | 677CC | –5.52±1.43 | –7.36±0.25 | 0.005 |
| *MTHFR* 2572CA+AA (n=10) | 677CC | –7.76±1.94 | –7.14±0.73 | 0.796 |
| *P* |  | 0.016 | 0.112 |  |
| *MTHFR* 2572CC (n=25) | 677CT+TT | –6.70±2.41 | –7.49±1.38 | 0.161 |
| *MTHFR* 2572CA+AA (n=5) | 677CT+TT | –12.61±7.18 | –11.58±6.03 | 0.905 |
| *P* |  | 0.061 | 0.159 |  |
| Abbreviations: MTHFR, methylenetetrahydrofolate reductase. Data were presented as mean ± standard deviation. MTHFR mRNA expression ratios were derived from –ΔC_T_ = – (C_T MTHFR_ – C_T 18S rRNA_). *P*-values were calculated by the Mann-Whitney test. | | | | |

| **Supplementary Table S11** AOR values of *MTHFR* 3'-UTR polymorphisms on CRC susceptibility according to *MTHFR* 677C>T genotypes | | | | | | | |
| --- | --- | --- | --- | --- | --- | --- | --- |
|  |  | CRC | | Colon | | Rectum | |
| Filter condition | SNP | AOR (95% CI) | *P* | AOR (95% CI) | *P* | AOR (95% CI) | *P* |
| None | *MTHFR* 677CT+TT | 0.92 (0.68–1.24) | 0.572 | 0.92 (0.65–1.30) | 0.631 | 0.94 (0.63–1.38) | 0.737 |
| None | *MTHFR* 2572CA+AA | 1.49 (1.10–2.03) | 0.010 | 1.40 (0.98–1.99) | 0.100 | 1.89 (1.27–2.80) | 0.008 |
| None | *MTHFR* 4869CG+GG | 2.17 (1.41–3.33) | <.001 | 1.77 (1.09–2.90) | 0.044 | 2.70 (1.59–4.59) | 0.002 |
| None | *MTHFR* 5488CT+TT | 1.66 (1.13–2.42) | 0.010 | 1.49 (0.96–2.31) | 0.100 | 2.02 (1.25–3.27) | 0.011 |
| None | *MTHFR* 6685TC+CC | 1.00 (0.70–1.44) | 0.999 | 0.97 (0.64–1.49) | 0.904 | 1.18 (0.74–1.87) | 0.562 |
| *MTHFR* 677CC | *MTHFR* 2572CA+AA | 1.20 (0.75–1.94) | 0.452 | 1.03 (0.60–1.78) | 0.914 | 1.55 (0.81–2.97) | 0.190 |
| *MTHFR* 677CC | *MTHFR* 4869CG+GG | 2.10 (1.16–3.83) | 0.015 | 1.75 (0.89–3.45) | 0.105 | 2.51 (1.17–5.41) | 0.019 |
| *MTHFR* 677CC | *MTHFR* 5488CT+TT | 1.42 (0.83–2.44) | 0.199 | 1.26 (0.68–2.34) | 0.463 | 1.83 (0.90–3.69) | 0.094 |
| *MTHFR* 677CC | *MTHFR* 6685TC+CC | 0.83 (0.49–1.38) | 0.468 | 0.77 (0.42–1.41) | 0.396 | 0.96 (0.49–1.89) | 0.910 |
| *MTHFR* 677CT+TT | *MTHFR* 2572CA+AA | 1.88 (1.21–2.92) | 0.005 | 1.75 (1.06–2.89) | 0.028 | 2.27 (1.31–3.94) | 0.004 |
| *MTHFR* 677CT+TT | *MTHFR* 4869CG+GG | 2.44 (1.26–4.74) | 0.008 | 1.87 (0.88–3.98) | 0.103 | 3.51 (1.59–7.77) | 0.002 |
| *MTHFR* 677CT+TT | *MTHFR* 5488CT+TT | 2.10 (1.17–3.78) | 0.013 | 1.92 (0.98–3.73) | 0.056 | 2.61 (1.26–5.39) | 0.010 |
| *MTHFR* 677CT+TT | *MTHFR* 6685TC+CC | 1.20 (0.68–2.12) | 0.533 | 1.18 (0.61–2.29) | 0.617 | 1.29 (0.64–2.62) | 0.476 |
| Abbreviations: *MTHFR*, methylenetetrahydrofolate reductase; CRC, colorectal cancer; AOR, adjusted odds ratio (adjusted by age, gender, hypertension, diabetes mellitus, body mass index, triglycerides, and high density lipoprotein-cholesterol); CI, confidence interval. | | | | | | | |
